# Supplementary material for: Functional Analysis of Autophagy-Related Gene ATG12 in Potato Dry Rot Fungus Fusarium oxysporum
Source: Int J Mol Sci. 2021 May 6;22(9):4932. doi: 10.3390/ijms22094932 (PMC8125257; doi:10.3390/ijms22094932)
Supplement: Supplementary file 1 [file ijms-22-04932-s001.zip › ijms-1164017-supplementary.pdf]

Supplementary information

Legends

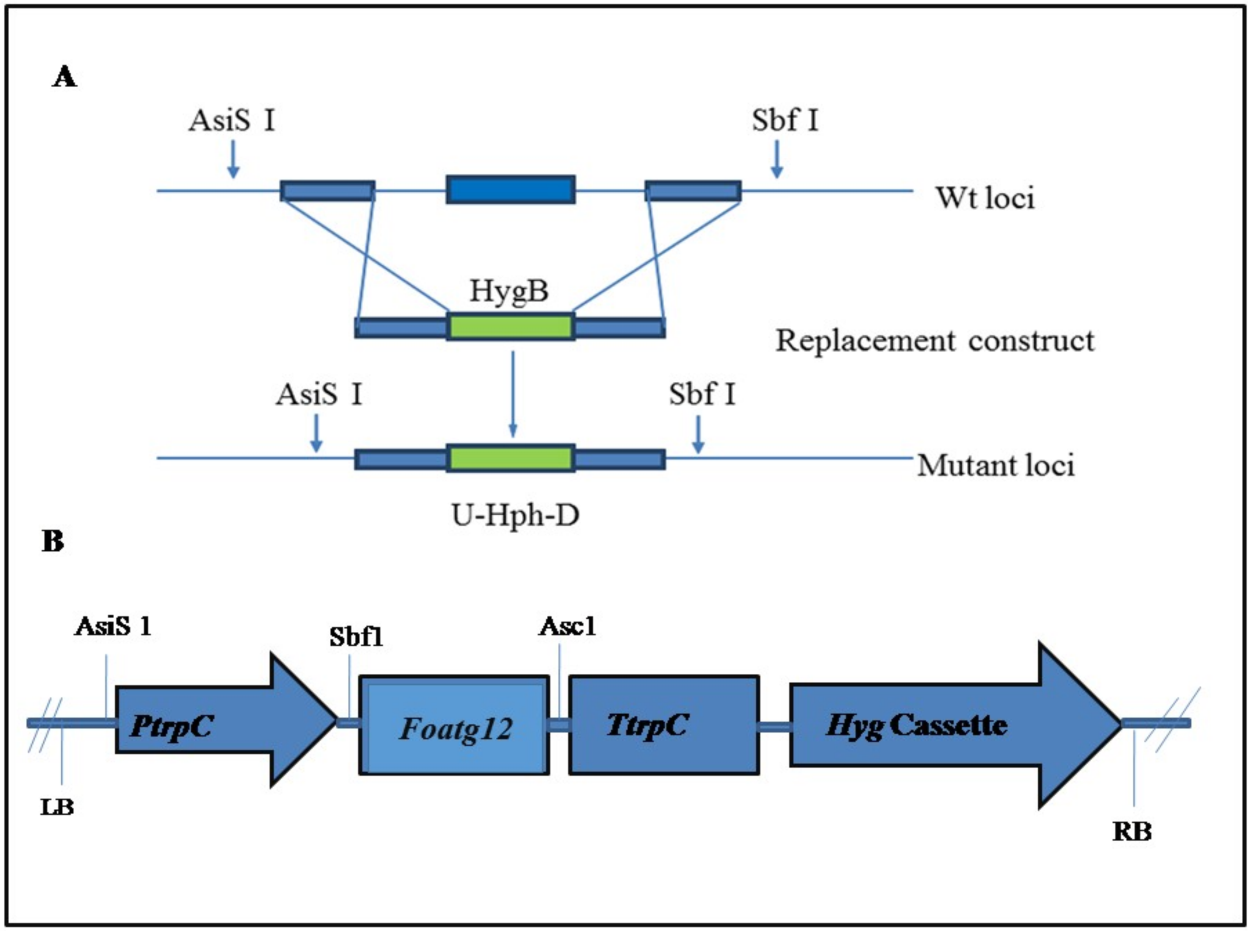

Figure S1. (A) The Foatg12 coding region was replaced with the HygB cassette. (B) The Foatg12 cDNA fragment was amplified with the indicated primers containing Sbf1 and Asc1 restriction sites inserted at 5' and 3' end of ORF respectively.

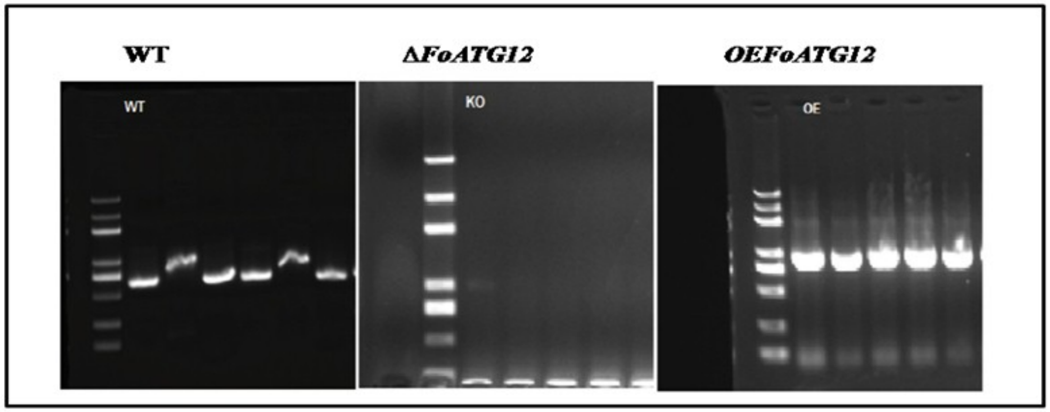

Figure S2. Molecular identification of mutants. Image represents expected shift in deletion mutant (Δ) and over expression mutants.

Table S1. Primers used in cloning and for qRT-PCR

Gene	Sequences 5` to 3`	
FoATG12 F	CCTGCAGG ATGTCAGACAACCCGCCACCAG	Cloning Primers
FoATG12 R	GGCGCGCC TCCGAAAGCAGGAGTGAGAGAG	Cloning Primers
Primer 5'UTR F	GCGATCGCACCATTCCATGCATTTGGCCTGGAGGACG	Deletion Primers
Primer 5'UTR-hph R	TGAGTTCAGGCTTTTTCATGGTGGCGGCCGCTGGCTTCGTTTCGTCTCGGTTC	Deletion Primers
Primer 5'UTR-hph F	GAACCGAGACGAAACGAAGCCA**GCGGCC**GCCACCATGAAAAAGCCTGAACTCA	Deletion Primers
Primer hph-3'UTR R	AGGCATACTGTAGATACCCCTGCAGGCTATTCCTTTGCCCTCGG	Deletion Primers
Primer hph-3'UTR F	CCGAGGGCAAAGGAATAGCCTGCAGGGGTATCTACAGTATGCCT	Deletion Primers
Primer 3'UTR R	CCTGCAGGCCCCACTACACCTCCATCTCGGCATAGCCAG	Deletion Primers
FOXG_VDH1F1	TCTCTACGGCACTTCTCAAT	qRT-PCR
FOXG_VDH1R1	TTGGTCAAGAATAGGCAGGA	qRT-PCR
FOXG_VMK1F1	CGATCACTCCATGTTCTGTC	qRT-PCR
FOXG_VMK1R1	GCTCCTGGATCAAGTAGACT	qRT-PCR
FOXG_ SNF1F1	CAAGATTGTTCACCGTGACT	qRT-PCR
FOXG_ SNF1R1	CGGGACCAGCATATAACTTG	qRT-PCR
FOXG_ NLP2F1	CATCCTTGCATCACCTATCG	qRT-PCR
FOXG_ NLP2R1	GATAGAGTGAGCCAGCTGTA	qRT-PCR
FOXG_ NLP1F1	GTCATGGCAGAGTACTTCAC	qRT-PCR
FOXG_ NLP1R1	ACAAAGTTGGCATCGATGAA	qRT-PCR
